# Parent-Mediated vs. Staff-Mediated Behavioral Models in Families of Autistic Children: A Comparative Study on Parental Stress, Co-Parenting, and Quality of Life

**DOI:** 10.3390/bs16030350

**Published:** 2026-03-01

**Authors:** Marco Esposito, Monia Trasolini, Roberta Fadda, Loredana Lucarelli, Marcella Caputi

**Affiliations:** 1 Department of Life Sciences, University of Trieste, Via Weiss 21, 34128 Trieste, Italy; marco.esposito@units.it; 2 Autism Research and Treatment Centre, Una Breccia nel Muro (UBNM), Via Soria 13, 00168 Rome, Italy; 3 Department XIV TSMREE of ASL ROMA1, Santa Maria della Pietà 5, 00135 Rome, Italy; monia.trasolini.aslrm1@nuovasair.it; 4 Department of Pedagogy, Psychology, Philosophy, University of Cagliari, 09100 Cagliari, Italy; robfadda@unica.it (R.F.); llucarelli@unica.it (L.L.)

**Keywords:** parent-mediated intervention, staff-mediated intervention, autism spectrum disorder, applied behavior analysis, parental stress, co-parenting, quality of life, co-parenting quality, family functioning, cluster analysis, early intervention

## Abstract

Background: Parental and caregiver stress represents a critical factor influencing family functioning and the quality of care in families of autistic children. Although previous research has identified multiple correlates of caregiver stress, the interplay between individual stress, co-parenting quality, and psychosocial contextual factors in families of autistic children remains insufficiently characterized within an integrated framework. Methods: This study adopted a cross-sectional exploratory design to examine stress levels, co-parenting quality, and associated psychosocial variables in caregivers. Standardized self-report measures were administered, and analyses included descriptive statistics, correlational analyses, and exploratory person-centered clustering procedures to identify patterns of co-occurring characteristics rather than discrete typologies. Results: Caregiver stress was significantly associated with indicators of co-parenting quality and psychosocial burden. Exploratory clustering analyses identified distinct patterns of caregiver experiences, characterized by differing levels of stress, perceived support, and co-parenting quality. These clusters should be interpreted as statistical profiles that reflect heterogeneous configurations of variables, rather than as stable or causal categories. Conclusions: Findings highlight the multidimensional nature of caregiver stress and underscore the importance of considering co-parenting quality and contextual factors within mental health promotion frameworks. The results are hypothesis-generating and support the need for future longitudinal and confirmatory studies to validate these patterns and clarify their implications for preventive and supportive interventions targeting families and care professionals.

## 1. Introduction

Autism Spectrum Disorder (ASD) is a complex neurodevelopmental condition characterized by persistent deficits in social communication and social interaction, alongside restricted and repetitive behavioral patterns that emerge early in life and often persist across development (DSM-5-TR^TM^; APA, 2022). Early clinical signs, including reduced eye contact, limited joint attention, repetitive behaviors, and atypical responses to sensory stimuli, are typically identifiable within the first two years of life. However, individual differences are evident in the atypical developmental trajectories of these children, which may vary substantially due to the spectrum’s substantial heterogeneity and environmental factors. These early-emerging characteristics are closely linked to difficulties in theory of mind, social cognition, executive functioning, and emotion regulation, all of which influence children’s adaptive and social competencies (Barklı & Doğan, 2025; Crowell et al., 2019). The complexity of ASD not only affects the child but also has extensive implications for the entire family system. Families raising autistic children frequently encounter challenges related to behavioral regulation, communication barriers, social misunderstanding, and difficulties in accessing adequate educational and therapeutic services (Saccà et al., 2019). These challenges often require parents to reorganize daily routines, modify developmental expectations, and manage increased caregiving demands that may intensify as the child grows older. Moreover, the child’s behavioral difficulties and support needs can disrupt family cohesion and contribute to emotional strain, which potentially influences both parenting practices and child outcomes.

A growing body of research emphasizes that ASD should be understood within a family systems framework (Downes & Cappe, 2021; Greenlee et al., 2018; Mazzoni et al., 2018; J. McHale & Dickstein, 2019; J. McHale et al., 2023; Oisakede & Cinnamond, 2026). According to family systems theory, families function as interdependent units in which changes in one member affect the entire system (Greenlee et al., 2018). In this perspective, parental stress, family adaptability, cohesion, communication patterns, and relational functioning critically shape the developmental context of autistic children. Evidence indicates that family-level variables such as structure, flexibility, emotional climate, and social support are significant predictors of child behavior, adaptive functioning, and socio-emotional development (Dunst, 2022; Greenlee et al., 2018; Oisakede & Cinnamond, 2026). These reciprocal and transactional influences underscore the importance of examining ASD not only as an individual diagnosis but also as a family-level condition that impacts multiple relational subsystems. Several systematic reviews have identified the centrality of family needs and unmet support requirements in shaping parental and child well-being (Dunst, 2022; Oisakede & Cinnamond, 2026). Unmet family needs, including insufficient informational support, limited access to specialized services, financial strain, and gaps in community resources, are strongly associated with increased parental stress, reduced coping capacity, and poorer psychological well-being. Likewise, families of autistic children frequently report difficulties navigating diagnostic pathways, fragmented services, and a lack of coordinated care, all of which can intensify parental burden. From a systemic viewpoint, the accumulation of unmet needs may not only reduce parents’ emotional resources but also affect family functioning more broadly, with implications for both parenting behaviors and child development (Dunst, 2022; Oisakede & Cinnamond, 2026).

Research examining parenting in ASD underscores that, although parents do not cause the disorder, their behaviors, emotional states, and interaction patterns play a meaningful role in children’s developmental trajectories (Downes & Cappe, 2021; Mazzoni et al., 2018; Oisakede & Cinnamond, 2026). Transactional models propose that children’s developmental difficulties can influence parental stress and responsiveness, which, in turn, may reinforce or mitigate children’s behaviors. Parents often describe feeling ineffective or uncertain about how to support their child’s communication, self-regulation, and social engagement (Crowell et al., 2019). At the same time, evidence suggests that supportive, structured, and responsive parenting strategies can promote child learning, enhance social communication, and facilitate emotional adjustment (Greenlee et al., 2018).

Overall, contemporary research highlights ASD as a condition that must be conceptualized within an ecological and systemic context, in which developmental outcomes are shaped not only by child characteristics but also by family processes, parental well-being, environmental resources, and broader contextual factors (Greenlee et al., 2018; Oisakede & Cinnamond, 2026). Consistent findings across reviews emphasize the importance of addressing family needs, supporting parents in their caregiving roles, and integrating family-centered approaches into intervention models (Downes & Cappe, 2021; Dunst, 2022; Karst & Van Hecke, 2012; Oisakede & Cinnamond, 2026). Understanding the general challenges and systemic influences that characterize families of autistic children provides a crucial foundation for examining more specific domains such as parental stress, quality of life, and co-parenting functioning.

### 1.1. Parental Stress and Quality of Life in Families of Children with ASD

Parents of children with ASD consistently report markedly higher levels of stress compared to parents of neurotypical children or those with other developmental disabilities (Davis & Carter, 2008). Parenting stress is conceptualized as the psychological and physiological response to the demands of raising a child when those demands exceed available resources (Abidin, 1992). In families of autistic children, stress arises from multiple, intersecting sources: the child’s behavioral challenges, communication difficulties, symptom severity, uncertainty about developmental milestones, and the demanding navigation of complex service systems (Bitsika & Sharpley, 2004; Ha et al., 2024). Across studies, more than 70% of mothers of autistic children report clinically significant stress levels, far exceeding normative thresholds (Kiami & Goodgold, 2017). A substantial body of research highlights child behavior problems as one of the strongest predictors of parenting stress. Externalizing behaviors related to sensory overload, meltdowns (Doherty, 2025), and non-compliance are consistently associated with higher parental stress both cross-sectionally and longitudinally (Barroso et al., 2018; Kanne & Mazurek, 2011; Suvarna et al., 2025). Indeed, autistic children frequently exhibit externalizing behaviors at higher rates than neurotypical peers, often due to sensory dysregulation, communication barriers, and daily demands that are misaligned with their developmental profiles. At the same time, internalizing symptoms such as anxiety or emotional withdrawal also contribute to elevated parenting stress, although these pathways are less studied (Bauminger et al., 2010). Longitudinal findings indicate that parenting stress consistently predicts increases in children’s externalizing behavior over time, whereas the reverse is less consistent (Rodriguez et al., 2019; Wright et al., 2023). While child-related factors have clear predictive value, parental psychological variables also play a critical role.

Parenting self-efficacy, defined as parents’ beliefs in their ability to manage parenting challenges, has been identified as a crucial mediator between child behavior problems and parental stress (Strauss et al., 2024). Higher self-efficacy is associated with lower parental stress, improved parental well-being, and fewer adverse emotional outcomes in both mothers and fathers (Kuhn & Carter, 2006; Hastings & Brown, 2002). Conversely, low self-efficacy can magnify parents’ perceptions of behavioral difficulties and increase stress reactivity (Firth & Dryer, 2013). Such mediating effects of self-efficacy may differ between mothers and fathers, underscoring the need for father-inclusive research and interventions. A growing number of studies have examined parenting practices as both outcomes and predictors of stress. High parenting stress is associated with more authoritarian and permissive parenting styles and decreased mindful parenting (Suvarna et al., 2025). These maladaptive patterns may, in turn, reinforce child behavioral difficulties, creating reciprocal escalations of stress within the family system (Mak et al., 2020; Osborne et al., 2008). Parenting stress is also linked to reduced use of effective coping strategies, limited emotional availability, and more frequent parent–child conflict, all of which undermine family functioning (Zaidman-Zait et al., 2017; Leonardi et al., 2021).

Beyond stress, parental quality of life (QoL) represents a broader multidimensional indicator of well-being. Studies consistently show that parents of autistic children experience lower QoL across physical, psychological, social, and environmental domains (McStay et al., 2014; Vasilopoulou & Nisbet, 2016). Contributors to reduced QoL include financial strain, time constraints, chronic emotional burden, and systemic barriers, such as poor access to specialized services (Catalano et al., 2018). Additionally, parenting stress and QoL are tightly intertwined: higher stress predicts lower QoL, and poor QoL exacerbates parents’ vulnerability to depression, anxiety, and burnout (Ni’matuzahroh et al., 2022). Recent research has begun to examine parenting burnout, a syndrome characterized by emotional exhaustion, detachment, and reduced parental accomplishment. Parents of autistic children show disproportionately high rates of burnout due to persistent caregiving demands, limited respite, and inadequate social support (Zhang et al., 2025). Stress is the strongest predictor of parenting burnout, and the two are linked through chain-mediating processes involving coping strategies and social support. High stress combined with low resources, such as inadequate social support, poor coping skills, or financial hardship, increases the risk of burnout (Falk et al., 2014). An essential factor shaping both stress and QoL is social support, which functions as a protective buffer against psychological distress. Social support is defined in literature as the perception or experience that one is loved and cared for by others, esteemed and valued, and part of a social network of mutual assistance and obligations; social support may come from a partner, relatives, friends, social and community ties (Phetrasuwan & Shandor Miles, 2009). Higher social support is associated with lower stress, improved mental health, and better family functioning (Phetrasuwan & Shandor Miles, 2009). Psychoeducational and skills-based parent training interventions have demonstrated effectiveness in reducing stress and improving coping and parental self-efficacy by increasing parents’ knowledge and confidence. Co-regulation skills and evidence-based knowledge of autistic children’s perspectives, gained through parent training, help parents manage their child and increase their sense of competence (Dillenburger, 2026; Jones & Prinz, 2005; Sofronoff & Farbotko, 2002). However, access to such interventions remains uneven across countries and socioeconomic groups, contributing to disparities in parental outcomes (Dawson-Squibb et al., 2020; Siller & Morgan, 2018). Overall, evidence converges on a clear conclusion: parental stress and QoL in families of autistic children are shaped by a complex interaction of child behaviors, parent psychological variables, environmental demands, and systemic factors. Understanding these interrelated influences is essential for designing adequate supports. Moreover, given the variability in how family systems function, research increasingly emphasizes the need to assess stress and QoL in relation to the specific intervention models families employ, particularly when parents serve as primary agents of intervention, rather than when trained professionals assume therapeutic responsibilities.

### 1.2. Co-Parenting in Families of Children with ASD

Co-parenting refers to how caregivers coordinate, support, and organize their shared parental roles, encompassing communication, mutual support, joint decision-making, division of caregiving responsibilities, and conflict management (Feinberg & Kan, 2008). In families of children with autism, co-parenting is especially relevant because the complexity of daily caregiving demands requires parents to collaborate effectively while adapting to high levels of stress, unpredictable behavioral patterns, and substantial service-related challenges. Research consistently shows that the presence of ASD in the family system influences parental interactions, role distribution, and the overall quality of co-parenting (Meirsschaut et al., 2010). Early studies (Crnic & Low, 2002; for a review, see Downes & Cappe, 2021) documented that parents of children with autism frequently experience discrepancies in perceptions of their child’s functioning, expectations for development, and confidence in their parenting abilities, which can influence co-parenting quality. Mothers, who often assume a disproportionate share of the daily caregiving load, report higher levels of stress and lower co-parenting satisfaction than fathers. This pattern reflects unequal division of labor, emotional burden, and time-intensive management of the child’s needs (Ozturk et al., 2014). These asymmetries can contribute to misalignments in parenting practices, reduced support exchanges, and increased relational tension, particularly when parents differ in their coping strategies or in their interpretations of the child’s behavior. A substantial body of evidence emphasizes the reciprocal links between child behavioral characteristics and co-parenting dynamics. More severe behavioral problems, particularly externalizing behaviors such as tantrums, aggression, and hyperactivity, are associated with lower co-parenting quality, greater parental conflict, and reduced cohesion in caregiving roles (Downes & Cappe, 2021; Karst & Van Hecke, 2012; Mazzoni et al., 2018; Oisakede & Cinnamond, 2026; Sikora et al., 2013). Internalizing symptoms, although less overt, are also associated with higher relational distress and less effective co-parenting coordination (Crowell et al., 2019). These findings support a transactional perspective in which child characteristics influence parental interactions, while parental dynamics, in turn, shape child outcomes through emotional climate, consistency, and the stability of caregiving patterns. The broader construct of family functioning is also strongly related to co-parenting; therefore, dimensions such as cohesion, flexibility, communication, and role clarity are significant predictors of both marital and co-parenting quality. Thus, families of autistic children tend to exhibit lower cohesion and flexibility and higher levels of disengagement and chaos compared to normative samples (Kostiukow et al., 2019). These family-level difficulties are closely tied to the intensity of the child’s needs and to chronic stress experienced by parents, which can reduce emotional resources for collaborative parenting. Studies show that stronger family functioning is associated with more adaptive co-parenting practices, fewer conflicts around caregiving roles, and more effective problem-solving in navigating the child’s challenges (Walton & Tiede, 2020). Relationship satisfaction represents another key component of the co-parenting system. Parents of autistic children frequently report reduced marital satisfaction, which is associated with higher parenting stress, diminished emotional intimacy, and limited time together due to caregiving demands (Sim et al., 2016). On the other hand, marital satisfaction can also serve as a protective factor: higher satisfaction is associated with more supportive co-parenting, better communication, and lower perceived burden. Support from partners and extended family has been shown to buffer stress and enhance the quality of co-parenting, particularly when external resources (e.g., services, respite, therapy) are scarce (He et al., 2022). These findings underscore the importance of relational resilience and shared responsibility in mitigating the impact of ASD-related stressors.

Recent studies offer a more nuanced understanding of how co-parenting interfaces with family needs and ecological demands (J. McHale et al., 2023). Families of autistic children frequently report unmet needs related to diagnostic clarity, therapeutic support, financial strain, social isolation, and future planning (Downes & Cappe, 2021; Oisakede & Cinnamond, 2026). When parents perceive higher unmet needs, co-parenting tends to be more conflictual and less coordinated, reflecting the strain that systemic barriers impose on dyadic functioning. Furthermore, family needs and stress levels predict variations in co-parenting through mechanisms involving emotional exhaustion, reduced coping capacity, and discrepancies in perceived competence (Thullen & Bonsall, 2017). Parents’ perceptions of autism traits also shape co-parenting; for example, higher levels of autism-related characteristics in children, including social communication difficulties, sensory sensitivities, and rigidity, are associated with lower family functioning and more strained co-parenting interactions (Ten Hoopen et al., 2023). These effects are mediated by parental stress, highlighting how psychological burden contributes to discordant parenting approaches, increased conflict, and reduced collaborative functioning. Overall, evidence converges on the conclusion that co-parenting serves as a central mechanism within ASD family systems, influencing both parental well-being and child development. High-quality co-parenting, characterized by coordinated roles, supportive communication, and shared decision-making, acts as a protective factor, enhancing family resilience, buffering stress, and promoting adaptive functioning. Conversely, conflict-prone or poorly coordinated co-parenting exacerbates stress, reduces family cohesion, and may contribute to poorer outcomes for children.

### 1.3. Scope and Hypotheses

The aforementioned literature directly informs the rationale for the present study, which examines two applied behavioral intervention models: one parent-mediated and one clinician-mediated, placing parents in different positions within the caregiving system. Intervention providers were psychologists who held a master’s degree in behavioral interventions.

When parents are asked to assume primary responsibility for implementing behavioral intervention, co-parenting dynamics may become more salient, influencing how families distribute responsibilities, manage stress, and sustain daily routines. Conversely, when professional staff take a leading role, co-parenting may be shaped more by emotional support, quality of involvement, and shared understanding than by direct caregiving demands. Examining co-parenting within these contrasting models provides a critical opportunity to understand how intervention structures interact with family processes at the systemic level. Using an exploratory cross-sectional approach, we examined differences between groups in three key parental domains: parenting stress, co-parenting functioning, and parental quality of life. Because both groups received standard ABA before the study, the comparison was expected to reveal differences in family-level and relational variables rather than differences attributable to treatment history. Specifically, we focused on the following domains that play a central role in family functioning in the context of autism: emotional strain associated with caregiving; quality of everyday interactions between parents and children; degree of cooperation and mutual support among caregivers; and parents’ perceived quality of life. We hypothesized that families in which parents were directly involved in the therapeutic process might show a different pattern in how they perceive and experience their interactions with their child and their coordination with their partner. Fewer differences were expected in the overall quality of life. However, aspects of social participation were expected to be sensitive to the child’s developmental stage. Finally, we expected to find some influence played by the child’s domains on the parent variables, as the literature suggests.

## 2. Materials and Methods

An exploratory cross-sectional study design was adopted, with participants (parent couples) recruited from families of children with autism who were receiving ongoing ABA services through a well-known voluntary association in Rome. Recruitment followed a non-probabilistic, consecutive sampling procedure: all families entering or already receiving behavioral services during the data collection window were screened for eligibility. Inclusion criteria were: (a) child with a clinical diagnosis of ASD; (b) child aged between early preschool and early school age; (c) both parents available to complete questionnaires when appropriate; (d) participation in one of the two behavioral support models routinely offered by the service; (e) middle Social Economic Status. Exclusion criteria were: (a) the presence of medical or neurological conditions unrelated to ASD that could impair questionnaire reliability; (b) incomplete data on core variables; (c) low Social Economic Status. Parents signed a legal contract with the association, in which they consented to ethical guidelines and privacy information regarding the conduct of the current behavioral intervention and the collection, analysis, and dissemination of research data. A copy of the contract is available upon request. Finally, the behavioral intervention was conducted in accordance with ethical guidelines for implementing evidence-based practices and the decision-making framework (Slocum et al., 2014; Contreras et al., 2022), and participants were reminded of their right to withdraw at any time. Ethical approval was obtained from the Ethical Board of the University of Cagliari (Prot. n. 0024052). Data were stored in private archives. Participants’ names were anonymized using an ID code in a database, which we used for data analysis.

### 2.1. Behavioral Models

Families were assigned to one of two support modalities already implemented within the clinical service: Model 1 or Model 2 (described below).

Model 1: Parent-mediated intervention. In this support modality, parents were directly coached and actively involved in strategy implementation; it consisted of an early intervention delivered primarily by parents in the home environment, with continuous supervision, coaching, and generalization sessions conducted at the center. The structure and rationale of this model closely mirror the cross-setting parent-mediated program described by Fava et al. (2011), in which parents are trained to serve as the primary agents of change in the child’s natural environment. Parents received structured training in evidence-based practices through workshops, modeling, guided practice, and live supervision. The training focused on: discrete trial teaching (DTT), incidental teaching (IT), natural environment teaching (NET), prompting and fading procedures, functional behavior assessment, and management of challenging behaviors. After the initial center-based training periods, parents implemented approximately 10–12 h of intervention per week directly at home. They followed individualized targets, materials, and procedures designed by supervisors, and regularly submitted videos or participated in live observations for fidelity checks. The model alternated between center-based functional assessment and parent training phases and parent-led home treatment phases, precisely as described in the cross-setting structure (3 weeks center, 3 weeks home, 1 week re-entry). This ensured that the child acquired skills with professionals and generalized them with parents in real-life routines. Under this model, parents became the primary implementers of programs in daily routines (communication, play, self-help), with therapists acting as trainers and supervisors rather than primary providers. At home, parents used behavioral strategies embedded in play, daily living activities, shared routines, and functional communication opportunities to maximize motivation and spontaneity, consistent with NET and IT approaches. Parents received continuous supervision: weekly coaching sessions with the child’s therapist, systematic fidelity checks (data collection, DTT procedures, play facilitation, discrimination training), and periodic re-entry sessions in which parents demonstrated skills in vivo. This model placed strong emphasis on generalization across persons (staff and parents), contexts (center and home), and materials and routines. Because parents were highly involved, this model produced a naturally rich learning environment but also required substantial family engagement and active participation.

Model 2: Staff-mediated intervention. In this support modality, therapeutic staff acted as the primary agents of intervention, while parents received indirect guidance, delivered by trained ABA therapists at the treatment center or, more rarely, at home. Unlike Model 1, in which parents served as the primary intervention agents, Model 2 relied on professional therapists as the primary implementers, with parents in a limited, supportive role (one participation in therapy with a tutor and one supervision session a month). The structure of this intervention closely aligns with the standardized model implemented and extensively documented in Esposito et al. (2017). Core components included Center-Based or Home-based Treatment (6 h/week). Children received individualized one-to-one sessions with qualified behavioral therapists. Sessions were divided between DTT and NET in quasi-naturalistic settings. Each child followed an individualized program designed by a supervisor based on the ABLLS (Assessment of Basic Language and Learning Skills; Partington et al., 2018), targeting: communication, receptive and expressive language, imitation (with and without objects), attention and joint attention, social play, motor skills, and autonomy/self-help behaviors. Therapists taught multiple acquisition targets in each session, using prompting and fading, reinforcement schedules, and criterion-based mastery (defined as three consecutive sessions with unprompted accuracy above 88%). A supervisor provided staff with weekly 1-h direct supervision for each child to ensure fidelity of teaching procedures. Also, in contrast to Model 1, parents were not responsible for delivering the intervention at home, even when tutors provided therapy. Their involvement consisted of attending an initial parent training course (6 3-h units), observing sessions, and participating in approximately 2 h/week to support the maintenance and generalization of mastered targets. Parents were not required to implement structured programs at home, although they did practice generalization of already mastered skills. This ensured uniformity in the staff-mediated treatment structure. Teaching sessions followed a standardized format: 9 trials per acquisition target, a 3-s response latency window, a prompt-and-fade hierarchy, differential reinforcement, mastery trials, and maintenance trials were integrated into each session. Skills were tracked continuously using data sheets provided by supervisors. Finally, a total of N = 94 parents (half couples) who met eligibility criteria completed the assessment.

### 2.2. Instruments

At the time of data collection, measures regarding families and children’s developmental profiles were collected, including sex, age, number of brothers/sisters, parents’ age and education, marital status, children’s developmental quotient scores with Griffith Mental Scales (Taddei et al., 2023), presence of language, and levels of interaction with the Autism Diagnostic Observation Schedule (ADOS) in a clinical setting (Lebersfeld et al., 2021). Children’s behavioral difficulties were evaluated using the Autism Spectrum Disorder–Problem Behavior Checklist (ASD-PBC), an 18-item caregiver-report tool specifically designed for children with ASD (Mahan & Matson, 2011). Each item is rated on a 0–2 severity scale. The scale includes two clinically meaningful domains such as externalizing behaviors, capturing outwardly directed behaviors such as aggression, tantrums, self-injury, or property destruction (acceptable internal consistency, α = 0.66), and internalizing behaviors reflecting more inwardly directed difficulties, including stereotypies, unusual sensory-seeking behaviors, or inappropriate sexual behaviors (internal consistency was acceptable with α = 0.70). The Total Score, reflecting global behavioral dysregulation, showed good reliability (α = 0.82).

The co-parenting relationship was assessed using the 23-item Co-Parenting Scale (J. P. McHale, 1997), which focuses on how caregivers collaborate or struggle to collaborate in their shared parenting role. Items invite parents to reflect on everyday relational dynamics, such as “How often do you and your partner support each other in front of the child?” or “How frequently does your partner criticize your parenting choices?” The scale includes four domains with clear clinical relevance: Family Integrity reflects warmth, unity, and shared parental identity (e.g., we feel like a good team as parents; reliability in this sample: α = 0.74). Devaluation captures subtle or overt undermining behaviors between caregivers (e.g., my partner puts me down in front of the child; reliability: α = 0.70). Conflict focuses on open disagreements or tension during parenting tasks (e.g., we argue in front of the child about rules; reliability: α = 0.82). Finally, Reproach/Discipline Coordination involves the ability to remain aligned in managing rules and consequences (e.g., we speak with one voice about discipline; reliability was very low, α = 0.17), a pattern commonly seen in small or low-variance subscales.

Parenting-related stress was assessed using the 36-item Parenting Stress Index—Short Form (Abidin, 1990; Guarino et al., 2008). Items describe thoughts and feelings that parents may experience in daily caregiving, such as “I feel trapped by my responsibilities as a parent” or “My child rarely does things that please me”. Parents respond on a 5-point Likert scale. The subdomains capture a different facet of the parent’s experience, including Parental Distress (PD), which measures the parent’s personal feelings of overwhelm, lack of support, or reduced sense of competence (e.g., I feel I cannot handle things; reliability, α = 0.90). Parent–Child Dysfunctional Interaction (P-CDI) reflects the parent’s perception that interactions with the child are not reinforcing or do not meet expectations (e.g., my child doesn’t seem to enjoy being with me; reliability: α = 0.67). Finally, Difficult Child (DC) captures temperament and behavioral challenges that make the child hard to manage (e.g., emotional reactivity, irritability as my child cries or fusses more than most children; reliability: α = 0.81. A Total Stress score provides a global index, α = 0.91.

Lastly, parents’ perceived quality of life (QoL) was assessed using the WHOQOL-BREF (Skevington et al., 2004; Ilić et al., 2019), a 26-item instrument evaluating multiple life domains. Items ask respondents to reflect on their physical and emotional functioning, social relationships, and environmental conditions. Examples include: To what extent do you feel your life is meaningful? How satisfied are you with your personal relationships? How well are you able to get around? The four domains include Physical Health, covering energy, pain, sleep, mobility, and daily activities (reliability, α = 0.77). Psychological Health addresses positive and negative emotions, concentration, self-esteem, and body image (reliability, α = 0.72). Social Relationships includes satisfaction with personal relationships, support, and intimacy (reliability, α = 0.56), consistent with the known limitation of the 3-item structure. Environmental wellness encompasses financial resources, safety, access to health services, and the quality of the home environment (reliability, α = 0.81). These domains provide a clinically meaningful snapshot of parents’ overall well-being and environmental support. The research dataset was deposited in the association’s private archive and is available upon request.

### 2.3. Data Analysis and Preliminary Statistics

All analyses were conducted in Python (version 3.11.6), with α = 0.05. Data preparation and statistical procedures followed recommendations on covariate selection, assumption testing, and model specification. Regarding preliminary analyses and group comparisons, we examined whether the two intervention groups differed on demographic and developmental variables, including continuous variables (e.g., child age, developmental quotient), which were compared using Welch’s *t*-tests; ordinal variables using Mann–Whitney U tests; and categorical variables using χ^2^ tests. The only significant difference was in child age (Model ½ = 54/75 months; t = −8.61, *p* < 0.001), whereas no other demographic, developmental, or behavioral variables differed between groups (for all details, see Table 1). Therefore, child age was included as the only covariate in subsequent ANCOVA models because it represents a pre-treatment characteristic not influenced by intervention exposure. To examine whether the intervention model was associated with parental stress, co-parenting, and parental quality of life, we estimated separate one-way ANCOVAs for each outcome variable. For each ANCOVA, assumptions of normality, homoscedasticity, and homogeneity of regression slopes were evaluated and satisfied (all Shapiro–Wilk *p*s > 0.12; Levene’s *p*s > 0.15; Model × Age interactions ns). After adjusting for age, no significant effects of Model emerged for Parental Distress or Difficult Child (*p*s > 0.07). A small but significant Model effect was observed for Parent–Child Dysfunctional Interaction (PCDI), F(1, df) = 5.08, *p* = 0.026, η^2^*_p_* = 0.052, with parents in the parent-mediated model reporting slightly more positive interactions. Child age was also a significant predictor of P-CDI (*p* = 0.002). The most robust finding was the large Model effect for Co-parenting Devaluation, which remained significantly higher in the staff-mediated model even after controlling for age, F(1, df) = 138.17, *p* < 0.001, η^2^*_p_* = 0.606. No other co-parenting scales differed significantly between groups (*p*s > 0.29). No significant model effects emerged for the Physical, Psychological, or Environmental domains (QoL). Child age significantly predicted the Social QoL domain (*p* = 0.021), but the Model effect was not statistically significant (*p* = 0.077). All details are provided in Appendix A.

To investigate the associations among child characteristics, parental stress, co-parenting, and QoL that differed across the two behavioral models, we computed Pearson correlation matrices for both interventions. Across both models, externalizing behaviors were positively associated with parental stress, particularly P-CDI and Difficult Child. Internalizing symptoms showed similar, though slightly weaker, associations. In Model 2, child age was positively related to parental distress and conflict, whereas in Model 1, it was associated mainly with P-CDI. Developmental quotient demonstrated weak-to-moderate negative associations with stress and devaluation in Model 2 but fewer associations in Model 1. Full matrices are presented in Table 2.

**Figure 1:**
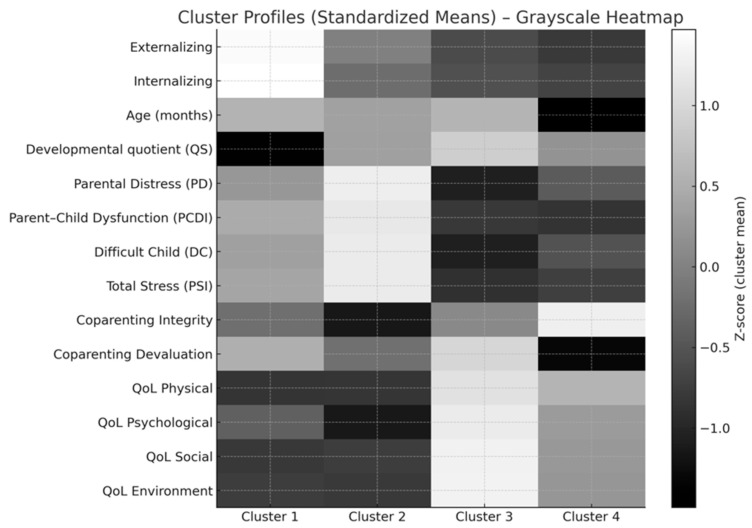
The heatmap displays the standardized mean scores (z-scores) for each input variable across the four clusters extracted using hierarchical agglomerative clustering (Ward’s method). Each row represents one of the variables included in the cluster analysis: child characteristics (Externalizing, Internalizing, Age in months, Developmental quotient), parental stress domains (Parental Distress, Parent–Child Dysfunction, Difficult Child, Total Stress), co-parenting dimensions (Coparenting Integrity and Coparenting Devaluation), and quality-of-life domains (Physical, Psychological, Social, Environmental). Each column represents one of the four clusters identified in the sample (Cluster 1–4). All variables were standardized before visualization to enable comparison across metrics with different scales. The heatmap uses a grayscale gradient, where lighter shades indicate cluster means above the sample average (positive z-scores), and darker shades indicate cluster means below the sample average (negative z-scores). Thus, lighter areas highlight dimensions that are particularly characteristic or elevated within a cluster, whereas darker areas indicate dimensions that are reduced or attenuated in comparison to the overall sample. This representation summarizes the distinct psychological and relational profiles emerging from the cluster solution. Cluster 1 shows lighter shades for Externalizing, Internalizing, Difficult Child, and Total Stress, and darker shades across all QoL domains, indicating a high-symptom, high-stress, low-quality-of-life profile. Cluster 2 shows lighter shades in all QoL domains and darker shades in stress-related variables, corresponding to a low-stress, high-functioning profile. Cluster 3 exhibits elevated levels of parental stress and Coparenting Devaluation, suggesting a high parental burden combined with strained co-parenting, particularly among families with older children. Cluster 4 shows lower Coparenting Integrity and moderate stress levels, indicating a younger-child, supportive coparenting profile, with a relatively preserved quality of life compared with Clusters 1 and 3. Because the heatmap uses standardized scores, shading differences reflect relative variation rather than absolute clinical thresholds. Variables were selected based on their conceptual relevance, empirical associations, and lack of redundancy, ensuring that clusters represent meaningful patterns in family functioning rather than background demographic differences. This visual summary supports the interpretation of the clusters as cohesive, theoretically grounded profiles of child symptoms, parental stress, relational dynamics, and well-being.

To derive multivariate family profiles, we evaluated all potential predictors such as child characteristics, parental outcomes, co-parenting scores, QoL domains, and socio-demographic variables using (a) theoretical relevance, (b) zero-order correlations, and (c) multicollinearity checks. Socio-demographic variables (parent sex, marital status, number of children, and education) showed inconsistent or negligible associations with parental outcomes and exhibited unbalanced distributions. These variables were therefore excluded to avoid spurious partitions. The final set of clustering variables included child characteristics (externalizing, internalizing, developmental quotient, age, sex), parental stress domains, co-parenting Integrity and Devaluation, and all four QoL domains. Consequently, a hierarchical agglomerative cluster analysis (Ward’s linkage, Euclidean distance) was conducted on standardized continuous variables; child sex was dummy-coded. Solutions with 2 to 5 clusters were evaluated using silhouette coefficients, cluster size balance, and clinical interpretability. Although the two-cluster solution showed the highest silhouette value, it yielded a highly unbalanced distribution. The four-cluster solution provided the best compromise between fit and interpretability, yielding four stable and clinically meaningful profiles (n = 16, 17, 22, 23) with a global silhouette of 0.212 and an R^2^ of 0.454. Cluster differences were validated using ANOVAs, which showed significant between-cluster variation across all major variables (*p*s < 0.001; the dataset is available on request). Cluster means and standardized profiles are visualized in Figure 1 (heatmap) and summarized in Table 3 (raw scores). These profiles captured distinct combinations of child symptoms, parental stress, co-parenting functioning, and QoL.

## 3. Results

Children in the parent-mediated group (Model 1) were significantly younger (M = 53.68 months, SD = 12.26) than those in the staff-mediated group (M = 75.00 months, SD = 12.25; t = −8.61, *p* < 0.001). This age imbalance justified including child age as a covariate in all subsequent ANCOVA models. No significant differences emerged in sex distribution or in the number of siblings. Developmental functioning, measured by the developmental quotient (QS), did not differ significantly between groups, although Model 2 showed slightly higher scores at the descriptive level (Model ½ = 60.3/67.3). Behavioral symptoms assessed through the ASD-PBC indicated comparable levels of externalizing and internalizing behaviors across the two models, with no significant differences. Parents in the two groups did not differ significantly in age (Model ½ = 40/39), sex distribution, marital status, or level of education. Global parenting stress (PSI-SF Total) did not differ between groups (Model ½ = 29.7/30.91; t = −0.64, *p* = 0.521).

Results indicate that among all demographic and developmental variables, only child age differed significantly. Consistent with the guidelines, age was used as the sole covariate in all subsequent ANCOVA models because it is a pre-treatment characteristic not influenced by participation in either intervention model. Regarding socio-demographic predictors of Parental Stress, co-parenting, and QoL, we examined whether key socio-demographic variables were associated with these outcomes independently of the intervention model. Contrary to common assumptions, mothers did not report higher stress levels than fathers. No PSI-SF domain differed significantly by parent sex (all *p*s > 0.20). For example, Total Stress did not vary between mothers and fathers, t(70) = 1.21, *p* = 0.230, d = 0.18, 95% CI [−0.10, 0.57]. Co-parenting dimensions also showed no significant differences, except for a trend toward lower Reproach among mothers, t(70) = −1.72, *p* = 0.090, d = 0.26. Quality-of-Life analyses revealed that mothers reported higher psychological QoL than fathers, t(74) = 2.11, *p* = 0.038, d = 0.41, 95% CI [0.03, 0.78], while no differences emerged in physical, social, or environmental domains. Interpretation: In this sample, being a mother was not associated with increased burden, but was associated with slightly better psychological well-being. Marital status was not associated with parental stress: all PSI indices showed nonsignificant differences between married and non-married parents (all *p*s > 0.20). Co-parenting scores were likewise unaffected. However, Environmental QoL differed significantly, with unmarried parents reporting higher scores (t(22.4) = −3.12, *p* = 0.003, d = 0.88, 95% CI [−15.6, −3.2]). Being married did not appear to be a protective factor against stress or co-parenting difficulties. The higher QoL among unmarried parents should be interpreted cautiously, given the small sample size. Parental education (low, medium, high) was not significantly associated with PSI stress domains (all *p*s > 0.10). However, education was meaningfully associated with co-parenting and QoL: Devaluation: F(2, 89) = 4.13, *p* = 0.019, η^2^ = 0.085. Higher education is perceived as greater Devaluation. Social QoL: F(2, 90) = 5.88, *p* = 0.004, η^2^ = 0.115. Lower education is associated with the highest social QoL. Environmental QoL: F(2, 90) = 4.10, *p* = 0.020, η^2^ = 0.083. Higher education was associated with lower environmental QoL. Higher education did not reduce stress; it was associated with more fragile co-parenting and lower social and environmental QoL. Number of children (0, 1, 2 siblings) did not predict differences in parental stress (all *p*s > 0.10). Significant associations emerged for Devaluation: F(2, 92) = 3.82, *p* = 0.026, η^2^ = 0.077. Lowest in families with one sibling; higher in families with 0 or 2. Psychological QoL: F(2, 92) = 3.11, *p* = 0.049, η^2^ = 0.064. Highest in families with zero siblings. Environmental QoL: F(2, 92) = 3.49, *p* = 0.035, η^2^ = 0.071. Highest in families with zero siblings. Having more children did not increase stress, but was associated with lower QoL and with greater perceived Devaluation in co-parenting. Child sex was one of the few sociodemographic variables to show consistent effects: Difficult Child: boys > girls, t(74) = 2.19, *p* = 0.032, d = 0.52. Total Stress: boys > girls t(71) = 2.07, *p* = 0.042, d = 0.48. Co-parenting Integrity: girls > boys. t(76) = 2.52, *p* = 0.015, d = 0.57. Parents of boys reported higher behavioral burden and higher total stress, consistent with known patterns of ASD externalizing behavior.

### 3.1. Correlational Analyses

Pearson correlations were computed among child characteristics (developmental quotient, externalizing and internalizing symptoms, age), parental stress domains, co-parenting scales, and quality-of-life scores. Table 2 reports the complete correlation matrix. Regarding child variables and parental stress, ASD-related Externalizing symptoms showed the strongest and most meaningful associations across the dataset. Externalizing was moderately to strongly correlated with all PSI stress domains, including Parent–Child Dysfunction Interaction (r = 0.42), Difficult Child (r = 0.33), and Total Stress (r = 0.37), indicating that disruptive and dysregulated child behaviors were closely linked to parental burden. Internalizing symptoms were also correlated with stress, although with slightly smaller coefficients. Internalizing was associated with Parent–Child Dysfunctional Interaction (r = 0.38), Difficult Child (r = 0.25), and Total Stress (r = 0.32), suggesting that anxious/withdrawn profiles also contribute to parental overload, albeit to a lesser extent than behavioral dysregulation. The Child Developmental Quotient (QS) showed moderate negative correlations with Externalizing (r = −0.50) and Internalizing (r = −0.38), indicating that lower developmental functioning was associated with greater behavioral and emotional symptomatology. Child age showed moderate correlations with Total Stress (r = 0.32) and, especially, with Coparenting Devaluation (r = 0.62), suggesting that families of older children experience greater fatigue and relational strain. Concerning parental stress and co-parenting, the PSI domains were highly interrelated, as expected for measures representing a unified construct. Total Stress showed strong correlations with Difficult Child (r = 0.92), Parent–Child Dysfunctional Interaction (r = 0.80), and Parental Distress (r = 0.74). Parental stress was also significantly linked to co-parenting dynamics. Total Stress correlated negatively with Coparenting Integrity (r = −0.52) and positively with Coparenting Devaluation (r = 0.52), indicating that higher stress is associated with reduced teamwork and greater perceived criticism or lack of support between partners. The strongest negative correlations in the entire matrix were observed between parental stress and QoL, especially: Psychological QoL (r = −0.54), Social QoL (r = −0.48), Environmental QoL (r = −0.42), Physical QoL (r = −0.48). These findings suggest that increased stress is accompanied by reduced emotional well-being, social support, and environmental satisfaction. Regarding child symptoms and family quality-of-life, externalizing behaviors showed substantial negative correlations with all QoL domains, most notably Physical QoL (r = −0.38) and Environmental QoL (r = −0.30). Internalizing symptoms displayed smaller but still meaningful negative correlations with Psychological QoL (r = −0.40) and Social QoL (r = −0.19). Together, these results highlight that both emotional/behavioral functioning and developmental level of the child are essential contributors to parental well-being. Co-parenting Integrity correlated positively with all QoL domains (e.g., Physical: r = 0.42; Psychological: r = 0.39), whereas Devaluation showed weaker, more variable associations. These patterns indicate that collaborative, cohesive co-parenting is a central protective factor, strongly related to physical and psychological well-being as well as environmental QoL. In sum, the correlational analyses reveal a coherent pattern in which child behavioral dysregulation (Externalizing) significantly contributes to parental stress and reduced family well-being. Internalizing symptoms contributes more modestly to stress and well-being. Parental stress is strongly associated with both co-parenting strain and lower QoL. Co-parenting Integrity emerges as a robust resilience factor across domains. These associations provided the rationale for selecting child symptoms, stress, co-parenting, and QoL variables as key inputs for subsequent multivariate models and cluster profiling.

### 3.2. Cluster-Based Family Profiles

The four-cluster solution revealed distinct family profiles based on child symptomatology, parental stress, co-parenting, and quality of life. The highest levels of child symptoms and parental stress were observed in families in Cluster 1 (n = 16; with a high proportion of male children). Children showed significantly elevated externalizing behaviors (M ≈ 6.0) and Internalizing (M ≈ 10.3) scores, and parents reported the highest Difficult Child and Total Stress scores (M TOT ≈ 109). Co-parenting was strained, with low Integrity (M ≈ 3.9) and high Devaluation (M ≈ 4.0). QoL was markedly compromised across all domains, particularly physical (M ≈ 45), psychological (M ≈ 46), social (M ≈ 43), and environmental (M ≈ 35), which were the lowest in the sample. Overall, Cluster 1 represents a high-symptom, high-stress, low-quality-of-life profile, indicating families under a substantial emotional and practical burden. Cluster 2 shows Low-stress, high-quality-of-life families (n = 17), in which children showed low levels of externalizing behaviors (M ≈ 1.4) and moderate levels of internalizing ones (M ≈ 4.8), with relatively high developmental quotients (M = ≈ 68). Parents reported the lowest stress levels (Total Stress M ≈ 67), and co-parenting was relatively cohesive, with high Integrity (M ≈ 5.6) and moderately elevated Devaluation (M ≈ 3.8). QoL was highest in this cluster across almost all its domains, especially physical (M ≈ 76), psychological (M ≈ 70), and social (M ≈ 71), with environmental QoL also in the upper range (M ≈ 62). This cluster can be described as a low-stress, high-quality-of-life profile, capturing families who manage the child’s condition with relatively fewer symptoms and stronger perceived resources. Cluster 3 included older children, high stress, and co-parenting strain (n = 22) with moderate externalizing (M ≈ 2.5) and Internalizing (M ≈ 4.8) scores. Despite symptom levels that were not as extreme as Cluster 1, parental stress remained very high (Total Stress M ≈ 105), and co-parenting was particularly strained, with the highest Devaluation scores in the sample (M ≈ 4.9) and relatively low Integrity (M ≈ 4.6). QoL in this cluster was intermediate: better than Cluster 1 but clearly worse than Cluster 2 (e.g., Physical QoL M ≈ 64; Psychological M ≈ 57). Most children were boys, and a large majority of these families belonged to the staff-mediated model (Model 2) (19 out of 22). This profile reflects families of older autistic children who experience persistent stress and a sense of being devalued or unsupported in their co-parenting relationship, despite symptom levels that are not the most extreme. Finally, Cluster 4 (younger children, moderate stress, supportive co-parenting; n = 23), it grouped families of younger children (M age ≈ 54 months), with relatively low externalizing (M ≈ 1.4) and the lowest Internalizing scores in the sample (M ≈ 3.7), and good developmental functioning (M QS ≈ 66). Parental stress was in the moderate range (Total Stress M ≈ 85), lower than in Clusters 1 and 3 but higher than in Cluster 2. Co-parenting looked relatively adaptive, with high Integrity (M ≈ 5.2) and the lowest Devaluation scores (M ≈ 2.1). Quality of life indices were in the mid-to-high range (Physical M ≈ 63; Psychological M ≈ 56; Social M ≈ 55; Environment M ≈ 47). Notably, all families in this cluster belonged to the parent-mediated intervention model (Model 1) (23/23). This cluster can be described as a younger-child, moderate-stress, supportive co-parenting profile, suggesting that parent-mediated interventions may be particularly common or effective in families with this configuration. Rather than a single risk factor, our findings indicate that families of autistic children tend to cluster into distinct psychosocial profiles, which may interact with the type of intervention model (Table 3 and Figure 1 depict these clustering networks).

## 4. Discussion

The present study compared two behavioral support models for families of autistic children, one parent-mediated and one staff-mediated, to examine their differences in parental stress, co-parenting functioning, and quality of life. While the two groups were broadly comparable across most domains after controlling for child age, several theoretically meaningful distinctions emerged, offering insight into how the intervention’s structure and agent may shape parental experience. A key first finding concerns co-parenting devaluation, which was markedly higher in the staff-mediated model, even after adjusting for age (η^2^ = 0.606). This effect is substantial and suggests a relational vulnerability specific to families in which therapeutic responsibilities are held primarily by professionals. Devaluation captures parents’ perceptions of criticism, diminished competence, or lack of mutual support within the parenting partnership. Consequently, elevated scores in this domain may reflect feelings of exclusion from the intervention process or discrepancies in parenting expectations when parents are not centrally involved in treatment. Prior research has shown that parents’ sense of partnership and alignment is a critical buffer against stress and predicts better child and family outcomes (Meirsschaut et al., 2010; Walton & Tiede, 2020). The present results align with family systems theory, supporting the notion that therapeutic models may indirectly influence the couple’s relational climate by shaping how caregiving roles are distributed and perceived. Similarly, a second relevant finding concerns Parent–Child Dysfunctional Interaction, which was slightly more favorable in the parent-mediated group. This effect was modest but remained significant after adjustment for age. Parent-mediated interventions often emphasize contingencies, shared routines, and sensitivity to the child’s communicative signals, which may enhance parents’ perceptions of reciprocity and engagement with their child. Previous evidence suggests that parent–child interaction quality is linked to self-efficacy, stress, and parental attributions (Hastings & Brown, 2002; Kuhn & Carter, 2006). Thus, although both intervention models followed behavioral principles, differences in who implements strategies may shape how parents experience everyday interactions with their child, and their acquired competence during treatment in ASD education tout court. Across most stress and co-parenting domains, however, no significant differences were observed. This suggests that the two models do not inherently confer broad psychological advantages or disadvantages. Instead, differences appear to cluster around relational dimensions that are particularly sensitive to parental involvement and role definition. The absence of between-group differences in the Parental Distress and Difficult Child domains contrasts with studies emphasizing the benefits of parent involvement in reducing parenting stress; however, such effects are typically observed longitudinally, as parents acquire skills (Jones & Prinz, 2005). The lack of differences here is consistent with the cross-sectional nature of the assessments; parents may have received some form of support during both interventions.

Regarding age-related effects, particularly in co-parenting devaluation, total stress, P-CDI, and social quality of life, the findings replicate those indicating that the caregiving burden increases as children grow older due to escalating behavioral, social, and adaptive demands (McStay et al., 2014; Barroso et al., 2018). Older child age was strongly associated with higher devaluation, suggesting that as challenges intensify, strain within the parenting partnership may become more pronounced. These results underscore the need for developmentally informed support structures, especially for families of older children. Frequently, parents receive training in behavioral techniques for simple communication, skill acquisition, and play routines, but often lack competence in the analysis and management of challenging behaviors, especially when children are nearing pre-adolescence, when social pressure is evident. Therefore, their education and training are demanding and should be reinforced throughout the therapy cycle. In fact, the correlational analysis further reinforces the pivotal role of child externalizing behaviors, which were the strongest predictors of parental stress across all domains, consistent with large-scale meta-analytic evidence (Wright et al., 2023). Externalizing symptoms were also associated with lower quality-of-life scores, confirming that behavioral dysregulation has wide-ranging effects on family well-being. Internalizing behaviors showed similar, though weaker, associations. Notably, stress was negatively correlated with all quality-of-life dimensions, reflecting a well-established reciprocal relationship in which heightened stress erodes psychological, social, and environmental resources (Vasilopoulou & Nisbet, 2016; Ha et al., 2024).

The cluster analysis offered an additional layer of understanding, revealing four distinct family profiles characterized by unique combinations of stress, co-parenting functioning, child symptomatology, and quality of life. The distribution of intervention models across clusters was asymmetrical: one high-functioning cluster consisted entirely of parent-mediated families, whereas another, characterized by elevated devaluation and stress, was almost exclusively staff-mediated. This pattern aligns with the ANCOVA findings and suggests that the intervention agent may contribute to broader profiles of family functioning. Although cluster analysis here was exploratory, the consistency of profiles suggests an ecological interpretation: different intervention structures may be associated with distinct relational and psychological configurations. This represents a clinical insight: when supervisors use decision-making processes to adapt the intervention, even considering family resources and dynamics, they foster their children’s skills and behavioral adaptation. Taken together, these findings highlight that the agent of behavioral intervention matters. Although parent-mediated and staff-mediated models may be equally effective for child outcomes, they appear to foster different relational climates and parental experiences. On the one hand, parent-mediated models may promote stronger perceptions of mutual support and more positive parent–child interactions by encouraging active engagement, shared routines, and increased parental competence. On the other hand, staff-mediated models may alleviate task demands but risk fostering feelings of distance or diminished agency, which may translate into relational strain.

### Limitations and Future Directions

Several limitations should be considered. The study was cross-sectional and could not assess causality. Moreover, the groups differed significantly in child age, requiring statistical adjustment; residual confounding cannot be excluded. Also, measures relied on self-report instruments, which may be influenced by parental mood or expectations. Additionally, families self-selected into service types, potentially reflecting underlying differences in preferences, logistical constraints, or parental characteristics not captured in the dataset. Another limitation of the study is that we did not collect any data about parental diagnostic status. It might be that, being autistic themselves, some parents of autistic children might experience higher levels of stress due to their conditions. This is a confounding variable that needs to be considered in future studies (Adams et al., 2021; Pohl et al., 2020).

Despite these limitations, the study provides valuable insight into family-level processes within different behavioral support models. Clinically, the findings underscore the importance of incorporating relational and co-parenting considerations into service planning. Staff-mediated models may benefit from structured opportunities for parental involvement, communication, and shared decision-making to mitigate devaluation and parent exclusion in the interventions. Parent-mediated models may require additional scaffolding for families with high stress or low self-efficacy to prevent overload.

Future research should adopt longitudinal designs to track how these relational differences evolve and examine whether they influence treatment adherence, parental well-being, and child developmental trajectories. Integrating observational measures of parent–child interaction and co-parenting communication would also strengthen the ecological validity of findings. This study contributes to a growing recognition that autism interventions must be evaluated not only for their child-centered outcomes but also for their systemic effects on the family. Understanding how different models shape parental stress, relational functioning, and quality of life is essential for designing services that optimize both developmental progress and family well-being.

## 5. Conclusions

This study contributes to the growing literature on caregiver stress by offering an integrated, exploratory perspective on the relationships among stress, co-parenting quality, quality of life, and psychosocial factors in families with autistic children. Rather than proposing definitive classifications, the present findings illustrate the heterogeneity of caregiver experiences and suggest that stress-related outcomes are best understood as arising from the interaction of individual and contextual dimensions. The exploratory clustering analyses revealed patterns that may be useful for conceptualizing variability among caregivers in families with autistic children; however, these profiles should not be interpreted as stable typologies or causal mechanisms. Given the cross-sectional nature of the study, all associations must be considered correlational, and causal inferences are not warranted. From clinical and public mental health perspectives, the results emphasize the importance of family-centered, context-sensitive approaches to stress reduction that address both individual caregiver well-being and broader family relational dynamics. Interventions aimed at supporting families with autistic children and care professionals may benefit from acknowledging the diversity of stress profiles of these families rather than adopting one-size-fits-all models. Future research should employ longitudinal and confirmatory designs to test the stability and predictive value of the identified patterns and to explore their relevance for targeted preventive and supportive interventions. Finally, it is important to consider that autism is a polygenic condition. Maybe parents themselves may also be autistic, sometimes receiving a diagnosis later in life or never. Thus, parental stress could also be understood in light of this perspective (Adams et al., 2021; Pohl et al., 2020). By conceiving of caregiver stress within a multidimensional, exploratory framework, the present study lays the groundwork for more refined, evidence-based approaches to mental health promotion in families of autistic children and in their care contexts.

## Figures and Tables

**Table 1:** Comparison of means between groups.

Variable	Model 1	Model 2	Test	Statistic	*p*
**Child characteristics**					
Age child (months)	53.68 (12.26)	75.00 (12.25)	Welch *t*-test	t = −8.61	<0.001
Sex child (males/females)	44/6	44/4	Chi-square	χ^2^ = 0.07	0.790
Number of siblings	0.80 (0.57)	0.79 (0.71)	Welch *t*-test	t = 0.06	0.949
**Parent characteristics**					
Age parent	40.10 (5.75)	38.88 (3.87)	Welch *t*-test	t = 1.24	0.218
Sex parent (males/females)	25/25	24/24	Chi-square	χ^2^ = 0.00	1.00
Education (ordinal)	2.22 (0.68)	2.25 (0.53)	Mann–Whitney U	U = 1195	0.971
Marital status (married/no)	40/10	40/8	Chi-square	χ^2^ = 0.03	0.869
**Cognitive profile**					
Developmental Q.	60.29 (22.70)	67.27 (16.64)	Welch *t*-test	t = −1.69	0.094
**Problem Behaviors**					
Externalizing	2.32 (2.39)	2.92 (2.64)	Welch *t*-test	t = −1.17	0.244
Internalizing	5.32 (3.62)	5.63 (3.80)		t = −0.41	0.686
**Parent stress**					
Parental Distress (PD)	29.68 (8.64)	30.91 (9.87)	Welch *t*-test	t = −0.64	0.521
Par. Child Dif. Inter. (P-CDI)	25.90 (6.30)	25.60 (4.40)		t = 0.27	0.787
Difficult Child (DC)	32.06 (8.56)	29.91 (7.88)		t = 1.27	0.208
**Co-parenting Dimensions**					
Family Integrity	5.06 (1.36)	4.57 (0.66)	Welch *t*-test	t = 2.25	0.027
Devaluation	2.07 (0.84)	5.25 (0.99)		t = −16.54	<0.001
Conflict	2.85 (1.40)	3.04 (1.29)		t = −0.68	0.496
Reproach/Discipline	3.93 (0.92)	3.79 (0.81)		t = 0.77	0.441
**Quality of Life (QoL)**					
Physical	3.63 (0.47)	3.49 (0.63)		t = 1.22	0.224
Psychological	3.32 (0.48)	3.36 (0.60)	Welch *t*-test	t = −0.35	0.726
Social	3.25 (0.57)	3.30 (0.73)		t = −0.31	0.752
Environment	2.78 (0.59)	3.03 (0.61)		t = −2.02	0.047

Note. Continuous variables were compared using *t*-tests to account for potential heterogeneity of variances. Ordinal variables (e.g., parental education) were compared using Mann–Whitney U tests. Dichotomous variables (e.g., sex, marital status) were compared using chi-square tests. Child age differed significantly between groups and was therefore included as a covariate. Values are reported as mean (standard deviation) unless otherwise specified. Model 1 = parent-mediated intervention; Model 2 = comparison model. All variables in this table represent psychological constructs that are primary outcomes of the study (child behavioral problems, stress domains, co-parenting dimensions, and quality of life).

**Table 2:** Correlations.

Predictors	Model	PD	P-CDI	DC	T. Stress	Integrity	Devaluation	Conflict	Physical	Psychol.	Environment
Age child	M1	−0.04	0.36 **	0.04	0.17	0.03	0.23	−0.02	−0.03	0.04	−0.06
Age child	M2	0.30 *	0.24	0.26	0.31 *	−0.38 *	−0.16	0.49 ***	−0.04	−0.15	−0.01
Dev. Quotient	M1	0.04	−0.37 *	−0.11	−0.19	−0.05	−0.22	0.06	−0.04	−0.14	−0.07
Dev. Quotient	M2	−0.12	−0.16	−0.07	−0.13	−0.10	−0.43 **	−0.15	0.21	0.23	0.34 *
Externalizing	M1	0.04	0.54 ***	0.34 *	0.43 **	−0.14	0.05	−0.11	−0.14	−0.02	−0.20
Externalizing	M2	0.32 *	0.29	0.39 **	0.29	−0.14	0.04	0.04	−0.54 ***	−0.36 *	−0.49 ***
Internalizing	M1	0.14	0.34 *	0.32 *	0.25	−0.01	0.17	0.01	−0.03	0.07	−0.02
Internalizing	M2	0.19	0.15	0.30	0.23	−0.17	0.05	−0.20	−0.47 **	−0.34 *	−0.45 **

Note. Pearson correlations are reported separately for Model 1 and Model 2. Model 1 = parent-mediated intervention; Model 2 = staff-mediated intervention. PD = Parental Distress; P-CDI = Parent–Child Dysfunctional Interaction; DC = Difficult Child; T. Stress = Total Stress; Integrity = Coparenting Integrity; Devaluation = Coparenting Devaluation; Conflict = Coparenting Conflict; Physical = Physical QoL; Psychol.= Psychological QoL; Environment= Environmental QoL. Correlations marked with *p* < 0.05 (*), *p* < 0.01 (**), or *p* < 0.001 (***) indicate statistical significance. Two-tailed tests were used. Missing data were handled using pairwise deletion. We removed Reproach (Coparenting) and the Social domains (QoL) due to low internal reliability.

**Table 3:** Clustering.

Variable	Cluster 1 (n = 16)	Cluster 2 (n = 17)	Cluster 3 (n = 22)	Cluster 4 (n = 23)
Externalizing Behaviors	6.03 (3.2)	1.41 (1.6)	2.46 (2.6)	1.41 (1.6)
Internalizing Behaviors	10.31 (3.4)	4.82 (2.9)	4.82 (3.1)	3.73 (2.4)
Age child	63.0 (21.4)	54.8 (17.1)	79.1 (28.2)	54.5 (16.6)
Developmental quotient	50.5 (18.2)	68.4 (21.0)	61.8 (17.3)	66.1 (12.7)
Sex child (% male)	87%	76%	81%	70%
PD—Parental Distress	39.6 (8.1)	27.0 (5.1)	38.8 (7.0)	33.0 (6.1)
PCDI—Dysfunctional Interaction	29.1 (6.3)	20.6 (3.4)	30.0 (6.0)	23.3 (4.6)
DC—Difficult Child	40.0 (7.2)	20.2 (5.8)	36.4 (8.0)	28.9 (6.0)
Total Stress (PSI)	108.8 (13.4)	67.4 (11.6)	105.1 (12.7)	85.1 (10.8)
Coparenting—Integrity	3.87 (1.1)	5.56 (0.9)	4.57 (1.0)	5.18 (0.8)
Coparenting—Devaluation	3.98 (0.9)	3.76 (0.8)	4.86 (1.0)	2.09 (0.8)
QoL Physical	45.5 (11.2)	76.0 (13.3)	64.4 (13.0)	63.0 (10.5)
QoL Psychological	46.0 (9.3)	69.6 (11.4)	57.3 (14.8)	56.2 (10.4)
QoL Social	43.2 (13.8)	70.7 (18.2)	55.6 (18.4)	55.4 (12.0)
QoL Environment	35.1 (10.9)	62.1 (12.4)	48.6 (14.1)	47.0 (8.8)

Note. Values are means with standard deviations in parentheses. All variables were standardized before clustering; however, raw scores are reported here for interpretability. Cluster 1 = High-symptom, high-stress, low-QOL Cluster 2 = Low-symptom, low-stress, high-QOL. Cluster 3 = Older child, high-stress, high devaluation. Cluster 4 = Younger child, moderate stress, supportive coparenting.

## Data Availability

Data are unavailable due to privacy or ethical restrictions; however, access is available upon request.

## Appendix A

Appendix A summarizes the ANCOVA models conducted for each outcome variable, adjusting for the significant difference in child age. These analyses provide age-corrected estimates of between-group differences and support the robustness of the primary findings.

**Table A1:** Results of ANCOVA models comparing parental stress, co-parenting, and quality of life between the two intervention models, adjusted for child age. Higher co-parenting devaluation in Model 2 persisted after adjustment and showed a considerable effect size (η^2^*_p_* = 0.606), indicating a substantial relational difference between groups independent of developmental factors. All other outcomes showed no differences in model fit after controlling for age.

Outcome	F (Model)	*p* (Model)	F (Age)	*p* (Age)	η^2^*_p_*
**Parent stress indexes**					
Parental Distress (PD)	0.12	0.731	1.64	0.204	0.001
P-C Difficult Inter. (PCDI)	5.08	0.026	9.76	0.002	0.052
Difficult Child (DC)	3.21	0.076	1.62	0.207	0.034
**Coparenting scales**					
Integrity	1.10	0.298	0.52	0.471	0.012
Devaluation	138.17	<0.001	0.17	0.679	0.606
Conflict	0.62	0.435	3.44	0.067	0.007
Reproach *	0.86	0.357	0.29	0.590	0.009
**Quality of Life domains**					
Physical	0.12	0.734	0.84	0.362	0.001
Psychological	0.31	0.577	0.20	0.656	0.003
Social *	3.20	0.077	5.54	0.021	0.034
Environment	2.98	0.087	0.10	0.753	0.031

Note. The table reports adjusted means and ANCOVA results comparing the two intervention models while controlling for child age, the only baseline variable that differed significantly between groups. Because all outcomes are adjusted for age, the effects shown in the table represent model differences as if the children in both groups were the same age, enabling an unbiased comparison of parental stress, co-parenting dimensions, and quality of life. For each outcome, the columns display the F statistic and *p*-value for the Model factor and the Age covariate, along with partial eta squared (η^2^*_p_*) as an indicator of effect size. Values of η^2^*_p_* closer to zero reflect negligible effects, whereas larger values indicate stronger associations attributable to the model rather than to chance or age-related variance. After adjusting for child age, most parental stress domains, specifically Parental Distress and Difficult Child, did not differ significantly between the two groups, indicating that the initial differences observed at the descriptive level were primarily attributable to developmental age rather than intervention structure. In contrast, Parent–Child Dysfunctional Interaction showed a small but significant model effect, reflecting slightly more positive interaction patterns among parents in the parent-mediated model. However, this domain was also strongly predicted by child age, suggesting that developmental demands influence parents’ perceptions of their interactions regardless of the model. The most notable finding concerns Co-parenting Devaluation, which remained markedly higher in the staff-mediated model even after controlling for age. This effect was substantial (η^2^*_p_* = 0.606), indicating a substantial relational difference between the models that developmental factors cannot explain. None of the other co-parenting dimensions showed significant group differences. Regarding quality of life, no model effects emerged in the Physical, Psychological, or Environmental domains. In contrast, the Social domain was significantly associated with child age but not with the intervention model, suggesting that social well-being declines with age, independent of treatment modality. Taken together, the table shows that after controlling for age, the two intervention models are broadly comparable across most psychological and quality-of-life indicators. However, Co-parenting Devaluation stands out as a robust and unique difference, indicating a higher perceived lack of support or undervaluation within the parenting partnership in families receiving the staff-mediated intervention. This suggests that differences between models are not general or widespread but are concentrated in specific relational dynamics closely tied to parents’ roles within the intervention. * The Reproach/Discipline Coordination subscale of the Co-Parenting Scale showed extremely low internal reliability (α = 0.17), indicating that scores were primarily driven by measurement error rather than actual variance. In contrast, the Social Relationships domain of the WHOQOL-BREF, which exhibited modest reliability (α = 0.56), was retained given the known limitations of its three-item structure; results for this domain should therefore be interpreted with caution. No other reliability issues were detected among the included variables.
